# The Use of Visualizations to Improve Bayesian Reasoning: A Literature Review

**DOI:** 10.3390/vision7010017

**Published:** 2023-03-02

**Authors:** Lucy Cui, Stephanie Lo, Zili Liu

**Affiliations:** Department of Psychology, University of California, Los Angeles, CA 90095, USA

**Keywords:** Bayesian reasoning, decision making, learning, visualizations, education

## Abstract

Decisions are often made under uncertainty. The most that one can do is use prior knowledge (e.g., base rates, prior probabilities, etc.) and make the most probable choice given the information we have. Unfortunately, most people struggle with Bayesian reasoning. Poor performance within Bayesian reasoning problems has led researchers to investigate ways to improve Bayesian reasoning. Many have found success in using natural frequencies instead of probabilities to frame problems. Beyond the quantitative format, there is growing literature on the use of visualizations or visual representations to improve Bayesian reasoning, which will be the focus of this review. In this review, we discuss studies that have found visualizations to be effective for improving Bayesian reasoning in a lab or classroom setting and discuss the considerations for using visualizations, paying special attention to individual differences. In addition, we will review the factors that influence Bayesian reasoning, such as natural frequencies vs. probabilities, problem format, individual differences, and interactivity. We also provide general and specific suggestions for future research.

## 1. Introduction

Bayesian reasoning has often been the focus of human statistical reasoning abilities. The need for it occurs in situations where there is prior evidence, and one must reason with the probability of two or more options given that evidence. Some examples of Bayesian reasoning include when a radiologist makes a diagnosis given mammogram results (a canonical mammography problem used to test Bayesian reasoning, e.g., [1,2]), when a jury makes a verdict given trial evidence, or when an ecologist identifies a species given its phenotypic traits. In these scenarios, the individual does not reach a conclusion of absolute certainty but a maximum likelihood. Through Bayesian reasoning, they can select the most probable choice given the data.

Yet, despite its usefulness and relevance to diverse situations, it has been shown that humans are, surprisingly, not as good at Bayesian reasoning as one’s exposure to situations that needs it would suggest. In fact, only 4% to 24% of students solve Bayesian problems correctly, which is considered very poor [3]. A common example of errors made in Bayesian reasoning is base rate neglect, in which the prior probability of an event is completely disregarded when comparing the posterior probabilities of two events, e.g., [4]. This mistake may occur because people often naturally perform Bayesian reasoning without the use of a mathematical formula. Ideally, one uses Bayes’ formula in their Bayesian reasoning: P(A|B) = [P(B|A)P(A)]/P(B), where P(A|B) is the posterior probability or the probability of an event given that event B has occurred, and P(B) is the prior probability [5]. However, teaching people to use Bayes’ formula for Bayesian reasoning has the difficulties of explaining the formula and mathematical notations and having students find the corresponding probabilities to match the formula’s components to understand their relations, e.g., [6]. Existing studies aim to identify ways to simplify this process for students.

Existing research approached the problem of poor Bayesian reasoning performance in two main ways: (1) using an evolutionarily-grounded format for the conditional probabilities: *natural frequencies* (10 out of 100 have an infection, 9 of those 10 tests are positive) instead of *probabilities* (probability of infection is 10%, probability of positive test given infection is 90%) and (2) providing visual aids to assist problem-solvers in seeing the relevant components of problems. The first approach aims to improve Bayesian reasoning by phrasing problems in a manner that would be most conducive to problem-solving, in which problem details can be more easily understood and calculated. For example, framing probabilities in terms of *natural frequencies,* such as 1 out of 100 instead of 1%, makes that quantitative information more concrete and readily computable. A recent meta-analysis [3] on the effect of natural frequencies compared to the (traditionally used) probabilities on Bayesian reasoning included 35 articles, with 226 performance estimates from the past 20 years of research. Their primary analyses showed support for natural frequencies over probabilities. Their moderator analyses (of many study characteristics) revealed short menu formats and visual aids as having the strongest moderation effects, improving performance for both natural frequency and probability problem formats.

The second approach aims to improve Bayesian reasoning by providing visualizations (e.g., bar graphs and icon arrays) as aids to help problem-solvers visualize important relations between problem details. The perceived benefit of visualizations is the translation of abstract information into a concrete form and the visual presentation of the components of Bayes’ formula. These visualizations could also help problem-solvers understand the relevance of certain problem components (e.g., prior probability) by displaying their relation to other problem components. In addition, visualizations provide a means for problem-solvers to visualize a posterior probability without getting stuck on a formula or calculation that may be difficult to formulate mentally. The aim is to make Bayesian reasoning easier for problem-solvers.

The goal of the current literature review is to examine the literature covering the use of visualization methods to improve Bayesian reasoning. Two similar, recent literature reviews [3,7] have included small sections on the topic of visualizations but not as the overarching theme or focus. Since the most recent review [3] on a similar topic (i.e., the use of natural frequencies vs. probabilities in Bayesian reasoning) was published in 2017, this literature review also serves as a detailed introduction to the use of visualizations to improve Bayesian reasoning and an update on the literature published on Bayesian reasoning since 2017 and includes 14 new publications not included in McDowell and Jacobs (2017) [3]. Additionally, we will be focusing on visualizations and providing a general overview of the other factors that influence Bayesian reasoning to provide context for understanding what has been carried out and what still needs to be carried out regarding visualizations to improve Bayesian reasoning.

## 2. Methods

Within this literature review, we have examined the effects of visualizations on Bayesian reasoning, specifically whether the use of visuals can improve the understanding of Bayesian reasoning and its performance within Bayesian problems. We broadly define visualizations as any visual aid, whether it be digital, on paper, physical, two-dimensional or three-dimensional, and static or interactive. We used the keywords: “bayesian reasoning” (alone) and in combination with “visual aid”, “visualization”, or “visual representation” to search Google Scholar and APA PsycInfo for relevant articles–last searched: 19 January 2023. To be considered for inclusion in this literature review, the articles needed to include experiments and use visualizations. Once the relevant articles were identified and checked for these criteria, we also checked the articles the authors cited and newer articles that cited the authors for any additional relevant articles we may have missed. Under the supervision of the first author, three research assistants worked independently to catalog these articles and keep the catalog updated. From the identified articles, we grouped articles into themes/subtopics and chose the most prominent articles to discuss for the larger groups.

**Figure 1:**
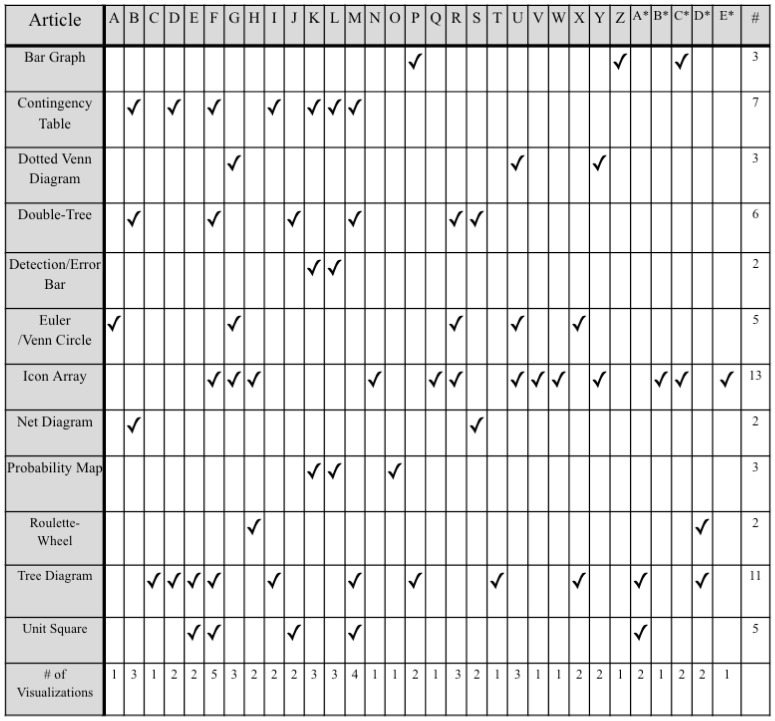
Types of visualizations used in articles. See Table 1 for key for article letters.

We will focus on discussing experiments that typically utilize one of two comparisons: (1) between a control group given no visuals or special teaching and an experimental group that is given visuals and/or taught how to use them, and (2) within subjects’ pretest and post-test results. Thus, this literature review was not meant to be an exhaustive review of all research on visualizations for Bayesian reasoning but one that focuses primarily on studies that fit these criteria. While many of these experiments used college students as participants, people from the medical field (doctors, medical students, patients), who need to use Bayesian reasoning in their life, was also common. See Figure 1 for overview of articles.

## 3. Visualizations That Have Been Effective

Many researchers have found visualizations to be an effective way of improving one’s accuracy in Bayesian problems. The research on this topic spans three decades. Among the pioneering researchers, Cole and Davidson (1989) [19] found that, when given either a contingency table (sometimes referred to as a 2 × 2 table), probability map, or detection bars (see Figure 1 for visual examples), the participants were significantly more accurate than baseline participants who did not receive a table or graphical representation. While participants in the control had an error rate of 16%, those who had some form of visualization had 5–7%. This study was carried out on a computer with digital visualizations, much like many of the studies after it, with the exception of some earlier work carried out on paper, e.g., [1], and some more recent work carried out on paper for classroom studies, e.g., [25] or in the field with doctors and patients, e.g., [22].

**Figure 2:**
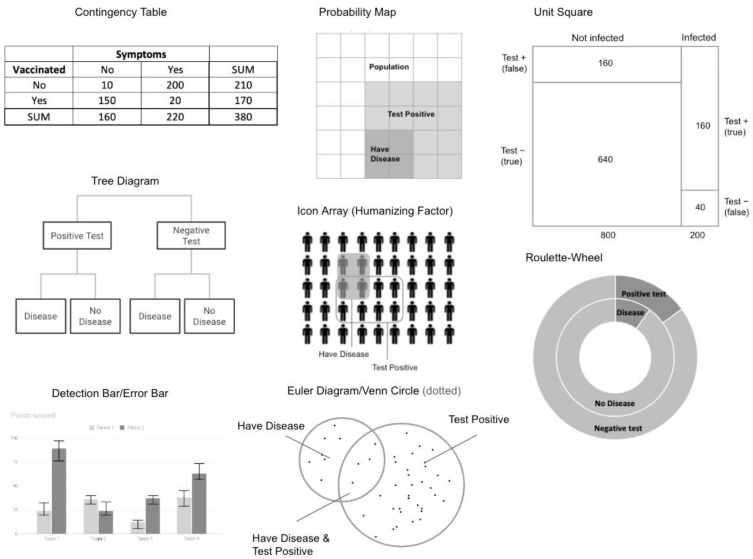
Examples of common visualizations used to improve Bayesian reasoning.

Many studies found certain visualizations to be more associated with higher Bayesian problem-solving accuracy, most notably icon arrays [13,14,25]. When comparing Bayesian performance between those who used an icon array and those who used a unit square (see Figure 2 for visual examples), Böcherer-Linder and Eichler (2019) found that participants with an icon array greatly outperformed those with a unit square. They speculated that having countable and discrete images (or icons) is beneficial in visualizing the given problems, thus leading to better Bayesian accuracy and decision-making. This finding was also substantiated by Brase’s studies [14,15]. In both studies, Brase consistently found that the participants were significantly more accurate when given icon arrays compared to having either no visual representation, Venn circles or roulette wheels [14,15]. The benefit of icon arrays has also been shown in children. Giegerenzer, Multmeier, Föhring, and Wegwarth (2021) [23] presented, for the first time, children’s ability to solve Bayesian problems using icon arrays. Most fourth graders and even most children with dyscalculia were able to solve Bayesian problems correctly (i.e., solving 50%+ of problems correctly) when using icon arrays.

A recent study using eye-tracking [28] provides a possible explanation for the effectiveness of icon arrays. They found that icon arrays make it easier to identify critical information for Bayesian reasoning and extract that information from text. The identification of critical information and the use of it for further problem-solving seems the most beneficial as participants’ interactions with the text and with the visualization were not affected by the mode of presentation (independently or together) of the materials.

Another potentially effective visualization method was the unit square with natural frequencies, as it could reveal relations in both a numerical and geometrical way [13]. However, this was shown to be less effective when compared to the use of an icon array and a 2 × 2 contingency table [13]. This study also found that double-tree diagrams were more effective when compared to tree diagrams, but this result has yet to be retested. More research may need to be carried out to compare these visualizations and their general effectiveness in promoting Bayesian reasoning understanding.

Beyond accuracy in Bayesian reasoning tasks, researchers have also looked at performance from an efficiency or response-time perspective. Binder, Krauss, Schmidmaier, and Braun (2021) [10] had physicians make Bayesian medical inferences with text only and with a tree diagram, and with problems worded in natural frequencies and probabilities. The physicians made inferences faster with a visualization (than without) and with a natural frequency format rather than the probability format. Diagnostic efficiency, which is measured by dividing the median time to make Bayesian medical inferences by the percentage-of-correct inferences, was best in the frequency tree condition.

## 4. Classroom Teaching of Visualizations to Improve Bayesian Reasoning

Multiple studies have also found increased Bayesian understanding and problem-solving accuracy through a combination of both classroom teaching and visualizations. Kurzenhauser and Hoffrage (2002) [25] compared the efficiency of two learning methods: rule training, in which participants were taught Bayes’s rule and how to use the formula, and representation-learning, where participants learned by doing (i.e., introduced a Bayesian problem, instructed on how to solve said-problem using a visual aid frequency-tree, and given multiple practice problems). Two months after the training, the students in the representation-learning group were almost three times more accurate than those in the rule-training group^7^.

Starns, Cohen, Bosco, and Hirst (2019) [31] also found benefits in classroom teachings using visualizations to improve Bayesian reasoning. Similar to Kurzenhauser and Hoffrage’s (2002) experiment above [25], after solving a pretraining problem, the participants watched a video about a visualization technique that used the length of bars to represent the probabilities within the problems, and were later given practice problems. They showed improved performance after the training, as well as evidence that this improvement was not explained by experiential learning from the problems [31]. Additionally, when replicated within a classroom setting with a shortened instruction, performance was also significantly increased, highlighting that even a shorter instruction can produce durable learning [31].

A recent study [17] tested the effectiveness of a Bayesian reasoning course designed based on the existing literature. The course used Bayesian problems written in natural frequencies, visualizations: unit square and double-tree diagrams, and a four-component instructional design (4C/ID: learning tasks, supportive information, procedural information, and part-task practice) model [37,38,39]. Medical and law students who took this Bayesian reasoning course improved their Bayesian reasoning skills [17].

## 5. The Creation of New Visualizations

In recent years, researchers have developed new visualizations to address the limitations of existing visualizations. For example, Binder, Krauss, and Wiesner (2020) [9] created net diagrams (*frequency net* and *probability net*) to address some of the limitations in contingency tables and tree diagrams. Contingency tables (or 2 × 2 tables) and tree diagrams are common visualizations used for supporting Bayesian reasoning, but they convey very different (and incomplete) information. The cells of the 2 × 2 tables contain *joint probabilities*, e.g., P(A∩B), like the probability that someone in the population is both sick and testing positive, but not *conditional probabilities*, e.g., P(A|B), like the probability that someone is sick given that they tested positive. On the other hand, the nodes of the tree diagrams convey conditional probabilities but do not display joint probabilities within the visualization. The *frequency net* displays absolute frequencies and all types of probabilities in its visualization. While the net diagrams did improve Bayesian reasoning, they did so at similar rates to the 2 × 2 tables and double trees. For conditional probabilities, frequency net diagrams and frequency double-tree diagrams yielded similar performances. When asked about joint probabilities, the participants performed best with probability 2 × 2 tables and probability net diagrams. While the net diagrams were not shown to be superior to existing visualizations, Binder et al. (2020) [9] did use insights from their study to give recommendations for teaching probability.

Starns et al. (2019) [31] presented a bar visualization technique inspired by a participant’s strategy in Gigerenzer and Hoffrage (1995, p. 695) [1], where two bars, each with a length that represents the probability of an event occurring and filled based on conditional probabilities, that help reasoners come up with the odds, instead of probabilities, for a Bayesian problem. The authors tested this bars visualization technique with solid fills (i.e., portion filled black with rest of bar unfilled) and a frequency format version (i.e., test results in either a continuous black or grey fill and discrete lines to convey the natural frequency component) and found that this visualization technique improved Bayesian reasoning, whereas the control groups showed no improvement. This study is unique in that the answers were requested in the form of odds (e.g., 1.5 times more likely) rather than probabilities.

## 6. Adding Interactivity to Visualizations May Be Helpful

Lastly, some evidence has shown that more interactive activities can also potentially aid Bayesian reasoning [40,41]. Tsai (2012) [40] found that interactive computer visualization substantially improved performance in both simple and more complex Bayesian reasoning problems. Additionally, when connecting the Bayesian problem to a real-time sporting event, participants who used a visualization not only performed better but also seemed to show less detrimental effects of overconfidence and showed more realistic internal reasoning accuracy (Tsai, 2012) [41].

Interactivity need not be digital. Interacting with physical objects also seemed to be useful. When participants were provided with a pack of custom-made playing cards that depicted the elements of the Bayesian problem and could be physically manipulated, Vallée-Tourangeau et al. (2009) [41] found that statistical reasoning in participants in the high-interactivity condition (i.e., a paper and pencil test with cards) was significantly better compared to those in the low-interactivity condition (just the paper and pencil test), regardless of any training or instruction and even with probability statements. Conversely, lower interactivity conditions resulted in stagnating performances among participants across three experiments [41]. Although these results may have been confounded by the uncontrolled amount of manipulation participants were able to manage, when researchers controlled this variable, they found that increasing the amount of interactivity offered by the cards was associated with a significant increase in performance ($M_{Low\;interactivity}$ = 0.52, SD = 0.35, $M_{High\;interactivity}$ = 0.77, SD = 0.31, *p* = 0.0098), thus demonstrating the strong relationship between physical interaction and Bayesian reasoning (Vallée-Tourangeau et al., 2009) [41]. Interactivity could also promote attention to and interest in the problem.

Adding interaction to static visualizations may not always be useful. Mosca, Ottley, and Chang (2021) [42] found that in some cases, adding interaction detracts from Bayesian reasoning performance, further noting that there are differences in how those with high versus low spatial ability respond to different interaction techniques and even the underlying base visualizations.

## 7. Some Mixed Results

However, not all researchers have found an effect using visualizations. When comparing the effects of icon arrays, Zikmund-Fisher et al. (2013) [36] found that, although icons seem to improve risk perception and recall, this may depend more on the individual’s graphical and/or numerical literacy skills than the format of the visualization. This could potentially be due to people with higher numerical literacy skills processing icon arrays by counting the number of icons, while those with lower numerical skills typically do not [36]. These results are further substantiated by Gaissmaier et al.’s (2012) [21] findings, who similarly found that the level of iconicity of the visualizations had no effect, but those with higher graphical literacy performed better. Similarly, Sirota et al. (2014) [30] found that iconicity had no effect on one’s understanding of Bayesian reasoning; namely, no positive effect of iconicity occurred for either pictographs or Euler circles. Although there are some concerns that this null iconicity effect might have resulted from an imprecise approximation of a population effect, further analysis found that this possibility could not be substantiated, giving relatively strong support to the finding that iconicity within visualizations failed to benefit statistical reasoning [30]. These results highlight that visualization may not be responsible for increasing Bayesian understanding, but rather an individual’s existing knowledge in understanding and deciphering graphics and numerical word problems. The idea that individual differences can contribute to Bayesian reasoning and the effectiveness of using certain visualizations will be discussed further in the next section.

Other studies have found that visualizations may have actually hindered participants’ accuracy in answering Bayesian questions [24,26]. In Khan et al. (2015) [24], only 20% of participants were able to correctly solve the mammography problem, and despite using visualizations, participants’ accuracy did not significantly improve. Similarly, although 89% of participants claimed to have used the visualizations while solving the Bayesian problems, some participants found that the visualizations were confusing and solely used the information provided in the text, while others doubted the credibility of the given graphics [26].

## 8. Consideration of Other Factors When Studying Visualizations

The mixed results discussed above stress the importance of considering other factors that may influence Bayesian reasoning in one’s study of visualizations. Listed in no particular order are the factors that should be taken into consideration when gathering insights from previous literature and when starting new research studies in this research area.

### 8.1. Natural Frequencies Versus Probabilities

A lot of research has found on Bayesian reasoning a positive effect of using natural frequencies (e.g., 57 out of 100 people have an infection) over probabilities (e.g., probability of infection is 57%). Gigerenzer and Hoffrage (1995) [1] were among the first to find this, with 48% of participants answering correctly with frequency-format Bayesian problems and 22% of participants answering correctly with probability formats. They propose that this preference for natural frequency formats is evolutionary in origin, as natural frequencies were used by humans long before probabilities, and thus, this numerical representation facilitates the computation of Bayes’ rule over probabilities [1]. This finding is substantiated by both Binder et al. (2015) [11] and Garcia-Retamero and Hoffrage (2013) [22]. When using natural frequencies in both visualizations (specifically 2 × 2 tables and tree diagrams) and written problems, participants’ performance was significantly higher (42%) compared to those who used probabilities (5%) [11]. The finding holds for problems without visualizations (26% correct inferences with natural frequencies vs. 2% correct with probabilities) and for problems with visualizations (51% correct inferences with natural frequencies vs. 6% correct with probabilities) [11].

The question of whether natural frequencies facilitate Bayesian reasoning more than probabilities has been studied in many populations: college students (discussed above), children, doctors, and patients. While children (fourth, fifth, and sixth graders) were unable (i.e., with 0% accuracy) to solve Bayesian problems when they were presented with probabilities, they were able to reach 19, 39, and 53%, respectively, when the problems were presented with natural frequencies [43]. In a study where an equal number of both doctors and patients made statistical inferences on the prevalence of disease and false positives, researchers found that performance in both groups was better when the information was provided in natural frequencies (51%) rather than probabilities (38%) [22]. Furthermore, a study on physician’s diagnostic inferences, evaluated based on positive predictive values (PPV) of four diagnostic tests, found that physicians correctly estimated PPVs more often when they were presented in the form of natural frequencies (46%) rather than probabilities (10%) [44]. More supporting research can be found in the meta-analysis comparing the use of natural frequencies to probabilities [3], though some null findings also exist, e.g., [34]. Wu et al. (2017) [34] found no difference in probability judgment accuracy across natural frequency and probability formats. It should be noted, however, that participants in this study also completed an information search task before judging the probability, which may explain the discrepancy between this study and others. Further, Wu et al. (2017) compared 14 different formats, which may have made it difficult to find a significant effect.

In comparison to Gigerenzer and Hoffrage’s (1995) [1] evolutionary argument on frequencies, Mellers and McGraw (1999) [45] theorize that frequencies help people to visualize “nested sets” (i.e., subsets within larger sets) while completing Bayesian tasks, thus also helping them to successfully solve the given problem. However, not all research has found such a significant effect on Bayesian reasoning for natural frequencies. Yamagishi (2003) [35] found that frequency format had a mild effect on Bayesian reasoning accuracy compared to using nested sets (i.e., diagrams like roulette wheels and tree diagrams that have this subset structure). However, the results demonstrated that the roulette wheel was more effective than the popular tree diagram. Eichler et al. (2020) [20] compared tree diagrams with unit squares and double-tree diagrams with 2 × 2 tables, choosing these pairs based on similar numerical information, and concluded that the characteristic of visualization to make the structure of the nested sets transparent was important in facilitating Bayesian reasoning.

Sirota et al. (2015) [46] also tested whether interpreting and visualizing Bayesian problems as a nested set would cue the relevant model and schemas to solve it (i.e., *problem interpretation hypothesis*) or if frequencies are easier to process and interpret (i.e., *format interpretation hypothesis*). Because the participants improved their performance due to set problem representations rather than frequency representations, Sirota et al. (2015) [46] concluded that the problem interpretation hypothesis is solely supported. In other words, having the proper problem mental representation is the key to improving Bayesian reasoning, not solely the format of the problem.

In contrast, other studies provided evidence that fit more consistently with the concept that the mind adapts most to work when using frequency information (*ecological rationality view*) compared to the nested set approach of Sirota et al. (2015) [46]. This *ecological rationality view* states that humans are best adapted to interpret frequencies due to their presence in the natural world, while the *nested set view* emphasizes the human ability to perceive and mentalize the nested set structure of the Bayesian problem [47]. When combining both pictorial representations with either frequencies or probabilities, researchers found that Bayesian problems with both pictures and frequencies led to the best performances, showing some support for the *ecological rationality view* [47,48]. However, it is not completely certain how these predictors interact with each other to procure these results. More research should be carried out on this topic.

### 8.2. Problem Format

Mismatches in problem presentation and question structure may produce more errors in Bayesian problems [49]. The relevant information for solving a Bayesian reasoning problem can be framed to be *condition-focused* (typical format, i.e., the base rate of having a condition/disease is the main focus) or *test-focused* (i.e., the base rate of a positive/negative test result is the main focus). Talboy and Schneider (2018) [49] compared a (*simplified*, i.e., minimal extraneous phrasing with visually offset information to show the nested structure of the problem) test-focused condition with three condition-focused conditions (*classic*: typical wording, *simplified*, and *probing*, asking four probing questions to highlight the four pieces of relevant information) and varied whether the response format was in frequency or percentage. Participants performed the best in the simplified test-focused condition, with performance in the frequency condition significantly higher than the percentage condition. On the other hand, participants performed significantly worse in the condition-focused conditions regardless of the numerical format [49].

### 8.3. Individual Differences

Along with these two previously mentioned factors (condition- or test-focused and the frequency or percentage formats), there are also many individual factors that can help or hurt one’s ability to solve and understand Bayesian reasoning problems. Two factors that are supported by research are one’s numerical and spatial ability [48,50]. Specifically, those with a higher numerical ability–or the ability to work with basic numbers–were more likely to perform better in Bayesian tasks compared to those with a lower numerical ability [7,48]. Meanwhile, one study showed that participants with low spatial ability were seemingly impaired by visualizations, while those with high spatial ability showed no effect from either pictorial representations or text displays [51]. Comparatively, Brase and Hill (2017) [48] concluded that spatial ability instead functioned more similarly to numerical literacy, with higher spatial ability more correlated with higher performance in Bayesian tasks. This conclusion is further supported because the spatial ability was still a significant predictor of performance with or without pictorial representations within the reasoning tasks [48].

However, the effect of these individual differences in numerical ability was found to diminish with simplified problem formats and natural frequencies, perhaps due to participants with a low numerate ability being more reliant on the external presentation of the problems to correctly process the relevant information [7]. In contrast, Brase and Hill (2017) [48] showed that both numerical and spatial ability contributions to Bayesian reasoning success were mainly independent of presentation format, as all possible simple interaction effects contributed to only 8% performance variance. More research should be conducted on this subject to see which simplifications and modifications would be helpful to those with low numeracy skills.

Another individual difference is strategy use. Individuals typically employ their own consistent strategy, commonly focusing on specific sources of information when solving Bayesian problems (e.g., relying on the true and false positive rates) [51]. Although some participants demonstrated clear strategies, others used strategies that could not be identified (27%), highlighting the variability between different people’s strategies [51]. This differential employment of strategies is also characterized by people’s gaze behavior, in which people who answered correctly visually focused on different aspects of the given representations compared to those who answered incorrectly [16,29]. Interestingly enough, despite mostly sticking to these consistent strategies, the majority of participants believed their performance to be poor (roughly 73%), revealing a discrepancy between their reliance on their individual strategy and their belief in its effectiveness [51], possibly they did not have a better choice in strategy.

## 9. Future Directions

Research on the use of visualizations to improve Bayesian reasoning is still in its earlier stages and is still growing. This paper reviews the existing literature and highlights areas that need more research to support the growth of this literature. We provide general suggestions as well as specific suggestions for each of these newer areas.

Our literature review identified two main considerations when evaluating the general effectiveness of visualizations for improving Bayesian reasoning and deciding whether to use them and which to use: (1) effectiveness may depend on individual differences, and (2) some participants do not use the visualizations because they find it confusing or untrustworthy, e.g., [26]. In order to address these two concerns, we suggest the collection of (1) individual difference measures, such as the numerical and spatial abilities discussed in this review, and (2) information regarding whether participants used the visualization and why they did not use it.

We also suggest the expansion of the dependent measures considered. The current literature includes some other dependent measures beyond just the accuracy of Bayesian problem-solving, including analyses of strategies used and gaze behavior [16,29,51]. However, the inclusion of such measures is far from the norm. The existing work that looks at these two measures has contributed key insights into this area. The analysis of strategy use identified an erroneous dependence of certain information (e.g., relying on true and false positive rates) [51]. The analysis of gaze behavior confirmed that visually focusing on relevant aspects of visualizations improves problem-solving accuracy [16,29]. Participants’ self-reported strategy use and gaze behavior (from eye-tracking) can help us understand how visualizations are used in problem-solving and whether certain patterns arise alongside individual difference measures, such as numerical and spatial ability. In addition, these dependent measures could also help piece apart the competing hypotheses and views, such as the *problem interpretation hypothesis* vs. *format interpretation hypothesis* and *ecological rationality view* vs. *nested set view,* when comparing different visualizations and arguing for why one visualization format may be more beneficial than another.

In addition to these dependent measures, we also recommend recording the frequency of each response (i.e., probability estimate from participants) for each problem and calculating the discrepancy of each response from the correct answer. The first recommendation is to identify common errors and whether a distribution of responses occurs, which allows for another metric to compare the effectiveness of different visualizations. Eichler et al. (2020) [20] serve as an example of identifying an error (e.g., selecting the wrong numerator) made by participants using a certain visualization (i.e., tree diagram) through the use of fraction responses instead of a probability response. The second is to identify whether over- or underestimating exists and whether it systematically varies with individual difference measures or the visualizations used.

In the realm of assessing and comparing the effectiveness of visualizations, we have a few specific recommendations:

1. **Exploring the interaction between certain visualizations and natural frequencies vs. probabilities.** There seems to be a potential for an interesting interaction between the use of (certain) visualizations and the use of natural frequencies vs. probabilities in the problem prompt. Some of the reviewed literature started investigating this possibility, but more studies that use either different visualizations or a wider range of visualizations are needed;
2. Narrowing down visualizations. As exemplified in Figure 1/Table 1, there are a lot of visualizations that are used in this research area, with some more popular than others. We recommend narrowing down the types of visualizations that are studied so that we can have more knowledge on a smaller set instead of less knowledge over a larger set, and even the standardization of the names of the visualizations (currently there are multiple names to refer to the same thing) to reduce confusion.

One way to narrow this down is simply to continue doing research on the visualizations that have been more frequently studied already. This seems like a completely reasonable approach and conserves time and resources in building a strong literature base. However, this approach may miss out on hidden gems (of insight). Another way to narrow it down might be based on reducing “redundancy.” For example, future studies can compare the visualizations that are similar in the presentation of information, and with enough evidence across the studies, a certain visualization can be ruled out. However, this approach faces the problems of publication bias for significant results and the need for the dedication of one research team or co-ordination across research teams;

3. **Understanding the importance of key features of certain visualizations.** We should be more deliberate in our comparison between different visualizations and choose visualizations (to include in studies) according to either testing competing hypotheses or identifying which features in those visualizations are helpful to students. For example, visualizations can be grouped based on whether quantitative information is presented in a discrete or continuous fashion or based on whether the quantitative information presented is a number, countable item, or area. These features can be used as an independent variable (e.g., area), and the visualizations (e.g., roulette wheel, unit square, probability map) can be chosen to match.

Another way to understand the importance of the different features of certain visualizations is to manipulate these visual features and see which version performs better. This ensures that quantitative information is presented in an optimal way for that particular visualization. For example, Witt and Dhami (2022) [33] found that icon arrays that placed “disease and tested positive” and “tested positive” areas near each other (proximal condition) enhanced risk judgments more than icon arrays that separated those two areas (distal condition). Whether a visualization is effective for Bayesian problem-solving could be dependent on how information is presented within these visualizations. Therefore, this is an important consideration to be aware of and something that is worth studying.

Regarding the factors that influence Bayesian reasoning, we recommend the extension of the literature on the factors listed in this review. The following are some more specific recommendations:

4. **Consideration of other problem formats.** Problem format is not commonly studied or manipulated alongside visualizations but should be considered. Whether problems are framed to be *condition-focused* (typical format, i.e., the base rate of having a condition/disease is the main focus) or *test-focused* (i.e., the base rate of a positive/negative test result is the main focus) seems to affect the solution rates across participants [49]. Some considerations are obvious, like making sure that the type of quantitative information (natural frequency vs. probability) matches the problem and the visualization. Bayesian reasoning is typically used in risk assessment and judgment, and with that, problems typically have a negative framing or very serious (health) outcomes. It would be interesting to see studies that manipulate positive vs. negative framing (e.g., test positive/negative vs. is healthy/ill) and see whether this influences over or underestimation. Cover studies of other more relatable contexts, such as social rejection/inclusion, could also be explored.
5. **Greater consideration of individual differences.** This literature review identified individual differences as a potential limiting factor for the effectiveness of visualizations. Future studies should investigate which simplifications and modifications (of visualizations and problem prompts) would be helpful to those with low numeracy skills. Additionally, other individual differences that could influence the effectiveness of visualizations or Bayesian reasoning, in general, should be explored. For example, things like risk aversion could bias probability estimates to be over or under the actual probability.
6. **Varying level interactivity and use with certain visualizations.** This seems to be the most lacking area of research. We can see this area of research going in two directions: (1) assessing a wide range of interactivity levels or comparing digital to physical manipulations and (2) including interactivity with each of the commonly studied visualizations.

This literature review presents a very exciting area of study that is still in its earlier stages and has many possible directions for future research. We suggest keeping up with the most recent publications and the consideration of your time, resources, and collaborators in your decision to take any of our suggestions.

## Figures and Tables

**Table 1:** References corresponding to articles outlined in Figure 1.

Reference ID	Reference
A	Benoy and Rodgers (2007) [8]
B	Binder et al. (2020) [9]
C	Binder et al. (2021) [10]
D	Binder et al. (2015) [11]
E	Böcherer-Linder & Eichler (2017) [12]
F	Böcherer-Linder & Eichler (2019) [13]
G	Brase (2009) [14]
H	Brase (2014) [15]
I	Bruckmaier et al. (2019) [16]
J	Büchter et al. (2022) [17]
K	Cole (1989) [18]
L	Cole & Davidson (1989) [19]
M	Eichler et al. (2020) [20]
N	Gaissmaier et al. (2012) [21]
O	Garcia-Retamero et al. (2013) [22]
P	Gigerenzer and Hoffrage (1995) [1]
Q	Gigerenzer et al. (2021) [23]
R	Khan et al. (2015) [2]
S	Kunzelmann et al. (2022) [24]
T	Kurzenhäuser and Hoffrage (2002) [25]
U	Micallef et al. (2012) [26]
V	Ottley et al. (2016) [27]
W	Ottley et al. (2019) [28]
X	Reani et al. (2019) [29]
Y	Sirota et al. (2014) [30]
Z	Starns et al. (2019) [31]
A *	Vogel & Böcherer-Linder (2018) [32]
B *	Witt & Dhami (2022) [33]
C *	Wu et al. (2017) [34]
D *	Yamagishi (2003) [35]
E *	Zikmund-Fisher et al. (2014) [36]

## Data Availability

No new data were created or analyzed in this study. Data sharing is not applicable to this article.
